# FAConstructor: an interactive tool for geometric modeling of nerve fiber architectures in the brain

**DOI:** 10.1007/s11548-019-02053-6

**Published:** 2019-08-11

**Authors:** Jan André Reuter, Felix Matuschke, Miriam Menzel, Nicole Schubert, Kévin Ginsburger, Cyril Poupon, Katrin Amunts, Markus Axer

**Affiliations:** 1 Institute of Neuroscience and Medicine (INM-1), Forschungszentrum Jülich GmbH, 52425 Jülich, Germany; 2 Cécile and Oskar Vogt Institute for Brain Research, University Hospital Düsseldorf, University of Düsseldorf, 40204 Düsseldorf, Germany; 3 Imaging and Spectroscopy Laboratory, CEA Saclay, Neurospin, Gif-sur-Yvette, France

**Keywords:** Nerve fiber structure, White matter phantom, Interactive visualization, Simulation model, 3D polarized light imaging

## Abstract

**Purpose:**

The technique *3D polarized light imaging* (3D-PLI) allows to reconstruct nerve fiber orientations of postmortem brains with ultra-high resolution. To better understand the physical principles behind 3D-PLI and improve the accuracy and reliability of the reconstructed fiber orientations, numerical simulations are employed which use synthetic nerve fiber models as input. As the generation of fiber models can be challenging and very time-consuming, we have developed the open source *FAConstructor* tool which enables a fast and efficient generation of synthetic fiber models for 3D-PLI simulations.

**Methods:**

The program was developed as an interactive tool, allowing the user to define fiber pathways with interpolation methods or parametric functions and providing visual feedback.

**Results:**

Performance tests showed that most processes scale almost linearly with the amount of fiber points in *FAConstructor*. Fiber models consisting of < 1.6 million data points retain a frame rate of more than 30 frames per second, which guarantees a stable and fluent workflow. The applicability of *FAConstructor* was demonstrated on a well-defined fiber model (*Fiber Cup* phantom) for two different simulation approaches, reproducing effects known from 3D-PLI measurements.

**Conclusion:**

We have implemented a user-friendly and efficient tool that enables an interactive and fast generation of synthetic nerve fiber configurations for 3D-PLI simulations. Already existing fiber models can easily be modified, allowing to simulate many different fiber models in a reasonable amount of time.

**Electronic supplementary material:**

The online version of this article (10.1007/s11548-019-02053-6) contains supplementary material, which is available to authorized users.

## Introduction

**Fig. 1:**
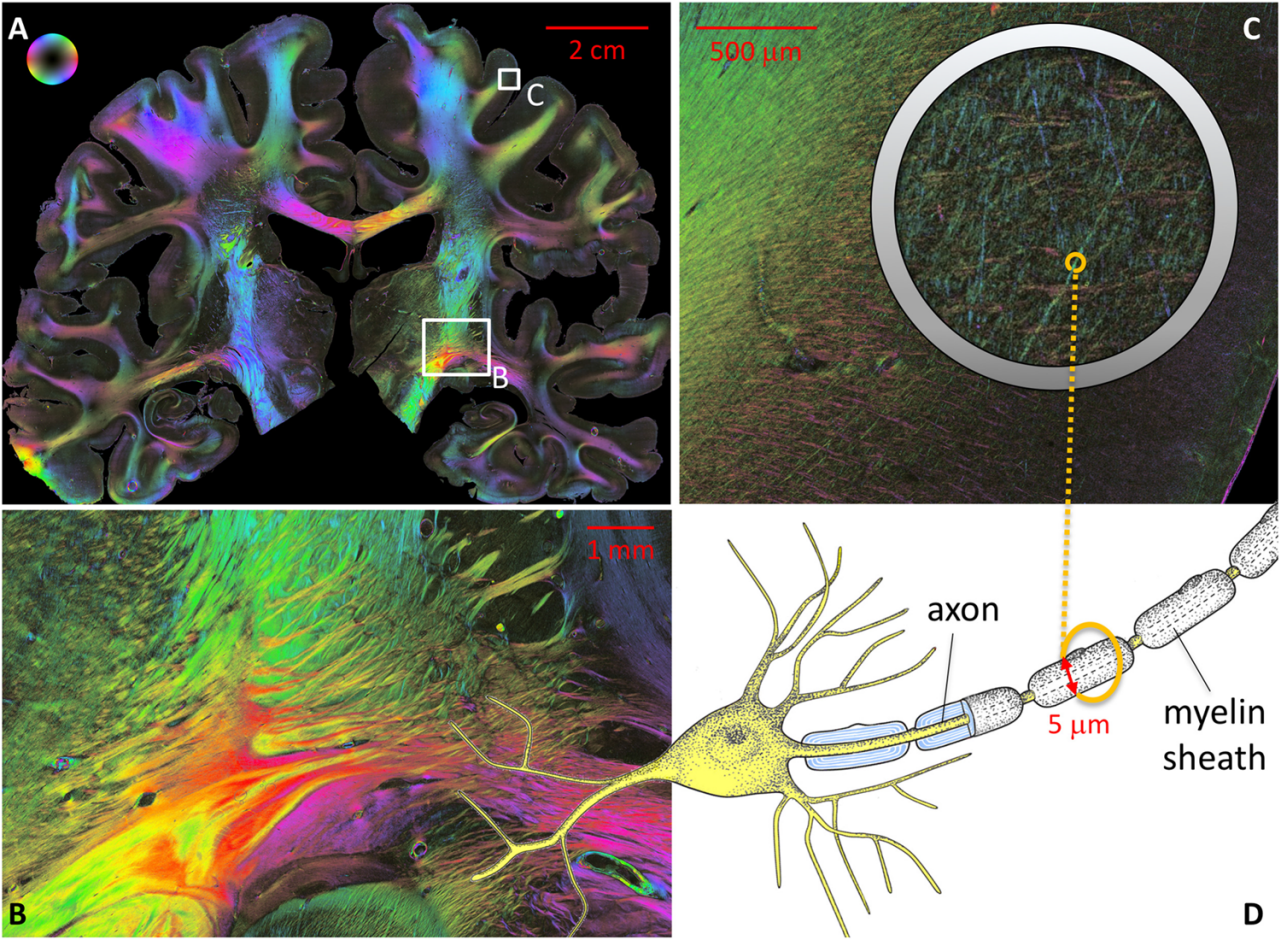
Fiber orientation maps of a coronal section through the human brain obtained with 3D-PLI. The determined nerve fiber orientations are color-coded as indicated by the color sphere on the top left. **a** Whole brain section with long- and short-range fibers at a glance. **b** Deep white matter fascicles of fibers (close-up of the region shown in **a**). The fascicle dimensions vary from mm to $\upmu \hbox {m}$ scales. **c** Cortical fiber tracts and individual fibers (close-up of the region shown in **a**). Typical fiber diameters lie below $10\,\upmu \hbox {m}$ [12]. **d** Schematic drawing of a neuron with a myelinated axon

Decoding the highly complex distribution and connectivity of neurons in the human brain is of major interest in neuroscience. Various neuroimaging techniques arose in recent years, each revealing specific characteristics of the brain’s nerve fiber architecture (e. g. individual myelinated axons, bundles of fibers, pathways) [3]. The most prominent techniques are diffusion magnetic resonance imaging (dMRI) [29], lightsheet fluorescence microscopy [26], (polarization-sensitive) optical coherence tomography [13, 27], and *3D polarized light imaging* (3D-PLI) [1, 2, 30]. None of these techniques has a real ground truth to compare with, leading indispensably to the idea of cross-modality research and technology-specific simulation approaches. Well-defined bio-mimicking numerical phantoms subjected to dMRI simulators have already been proven to be essential tools for better understanding of the underlying physical mechanisms (e. g. water diffusion, magnetic field dependencies) and, ultimately, improving the interpretation of the measured signals [7, 9, 10, 14].

In the present study, we focus on fiber modeling and simulation aspects of the microscopic neuroimaging technique 3D-PLI, which enables the reconstruction of the nerve fiber architecture of whole postmortem brains at micrometer resolution [1, 2, 30]. With 3D-PLI, the spatial orientations of fibers are derived from unstained histological brain sections in a polarimetric system that measures the local birefringence. This birefringence is mainly caused by the highly ordered molecular arrangement of the myelin sheaths wrapped around many axons in the white matter. Figure 1 demonstrates the unique potential of 3D-PLI to carve out the entire spectrum of real nerve fiber structures ranging from individual cortical fibers ($\upmu \hbox {m}$ scale) to long-distance fiber bundles or fascicles (mm to cm scales), even within one coronal human brain section. However, in brain regions where fiber structures show dispersed orientations within one volume element, the derived orientations might be misinterpreted. In order to underpin or improve fiber orientation estimations by means of 3D-PLI, two complementary numerical simulation approaches have recently been implemented: (i) the matrix calculus simulation (*simPLI*) [6, 16, 17] and (ii) the *finite-difference time-domain* (FDTD) simulation [18, 19].

Both types of simulation require numerical fiber phantoms as input, describing anatomical and optical properties and mimicking brain tissue to a certain degree of realism. In the present study, the focus was on white matter phantoms defined by numerous fiber models with specified fiber diameter, myelin thickness, spatial course, refractive indices, etc. The generation of such fiber models and resultant phantoms, however, is challenging and time-consuming, especially when targeting complex and large-scale models.

We introduce the easy-to-use open source software tool *Fiber Architecture Constructor (FAConstructor)* enabling fast and efficient generation of white matter phantoms. It allows to define *fiber objects* (e. g. individual fibers, tracts, bundles) which can be rotated, (re)moved, duplicated, and spatially integrated into complex large-scale phantoms. To enable modification of a large amount of fibers, fiber objects can be arranged in groups. By this means, the brain’s multiscale characteristics can be built from small numerical tissue units complying with the specific requirements posed by the different simulation approaches. The implemented graphical user interface allows the user to examine and interact with the generated fiber models in 3D space and provides direct visual feedback prior to simulation.

We demonstrate the applicability of *FAConstructor* by using the example of the so-called *Fiber Cup* phantom [7], which has initially been designed for an international competition on fiber reconstruction approaches (i. e. tractography algorithms) based on dMRI-like data. The resulting phantom is finally subjected to both the *simPLI* and the FDTD simulations.

**Fig. 2:**
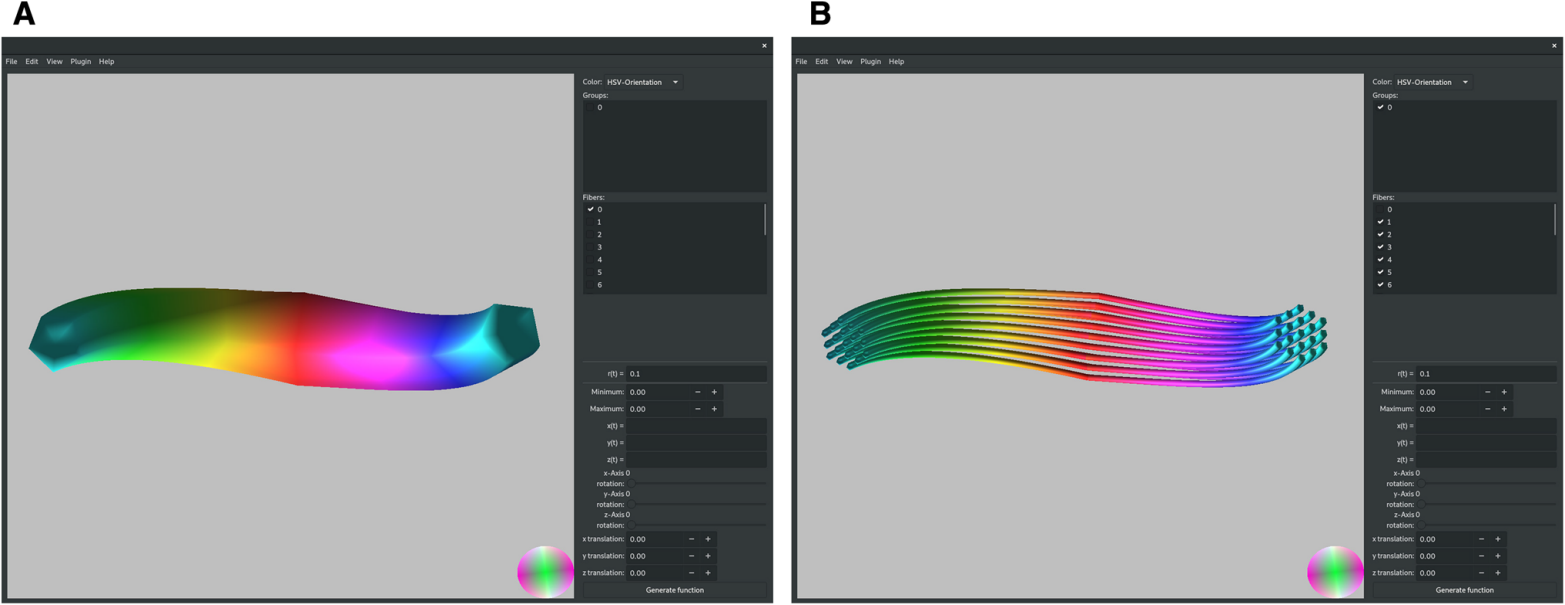
**a** Fiber object representing a fiber bundle (envelope) as generated by *FAConstructor*. The main monitor shows the fiber object from a certain view. Objects can be rotated, scaled, or translated by the user. Lists of all generated fibers and groups are shown on the top right. Objects can be selectively shown by the corresponding checkboxes next to their labels. The parametric functions describing the coordinates and radii of the displayed objects are also displayed. Here, the object has been generated by the parametric functions $\left( x(t) = 50 \cdot \sin (t\cdot \pi ),\ y(t) = 50 \cdot \cos (t\cdot \pi ),\ z(t) = 50\cdot t,\ r(t) = -\,|15 \cdot t |+ 15, t \in \left[ -\,0.5, 0.5\right] \right)$. All other options are accessible through the menu bar at the top. **b** Fiber object filled with fibers. With increasing radius of the fiber object, the distance between the fibers increases accordingly

## The fiber architecture constructor (FAConstructor)

The *FAConstructor* is a simple, effective, and interactive tool to generate and visualize fiber objects as input for simulations or similar purposes. An open source version of the code and a detailed description of compilation and usage are provided through the following GitHub repository: https://github.com/3d-pli/FAConstructor.

The tool allows an exact definition of curves using parametric functions or interpolation methods. Each curve contains a number of data points consisting of coordinates in three-dimensional space and radii for each data point. By this means, individual fibers or entire fiber bundles will be described in the following and referred to as *fiber objects*.

### Generation of fiber objects

A key feature of the *FAConstructor* is the generation of fiber objects with defined pathways and geometries. The user is able to define arbitrary fiber pathways using either interpolation methods with given coordinates or parametric functions. As the tool allows fiber objects to overlap, postprocessing might become necessary to avoid fiber intersections.

The manual input of three-dimensional coordinates allows a precise definition of fiber pathways. A different radius can be assigned to each coordinate, allowing each object to grow or shrink along its trajectory. In addition, the user can apply cubic spline interpolation to smooth the fiber object. In this case, both coordinates and radii are interpolated.

A major advantage of the developed tool is that individual fiber objects can be modified without need to regenerate the whole model. Both whole fiber objects and individual fiber coordinates can be rotated, translated, or duplicated. In this way, the user can modify an already existing fiber model and build large-scale models without much effort. Furthermore, it is possible to group several fiber objects together and change or duplicate them at the same time. The groups can be exported for future usage.

To facilitate the generation of nested fiber objects, the *FAConstructor* allows to fill a parent fiber object with multiple child objects, which can be filled by other fiber objects and so on. Here, we only consider fiber objects that were once filled with individual fibers, so we refer to the parent fiber object as fiber bundle and to the child objects as fibers.

When filling a fiber bundle with fibers, the cross section with minimum radius is used as starting point. The fibers are arranged on a triangular grid (see Fig. 2b), and the radius of every fiber and the minimum distance between neighbored fibers are defined by the user. The triangular grid is rotated from one data point to the next using the tangents of each point and computing the rotation angle using the Rodrigues formula [5].

If the radius increases along the course of the fiber bundle, the distance between the fibers increases accordingly. This guarantees that the fibers in the bundle do not intersect with each other, provided that the user-defined minimum distance does not fall below the diameter of the fibers. The generated fibers are stored as a group, allowing efficient modifications of the fiber bundle.

**Fig. 3:**
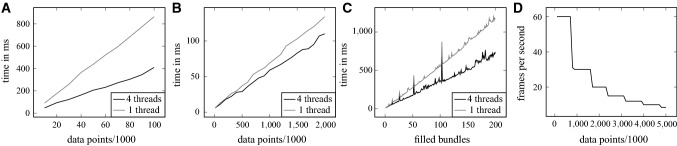
Performance measurements of four scenarios during the generation of a fiber object. The *x*-axis shows the number of data points used for the respective measurements, and the *y*-axis shows the elapsed time or the frame rate. The used parameters are shown in Eqs. 1–2: **a** Generation of fiber objects by manual input. The data points are defined and connected using cubic spline interpolation. **b** Generation of a helix using parametric functions. **c** Filling *n* fiber objects containing 1000 data points with 21 nested fiber objects each. The peaks in plot are most likely due to the memory utilization of the data type. **d** Frame rate while interacting with *n* fiber objects on screen

Coordinates and radii of the fiber objects are defined as single-precision floating-point values, which can be saved either in a hierarchical file system-like data format (HDF5, https://www.hdfgroup.org) or, alternatively, in a plain text file format. HDF5 is advantageous, because it allows to handle metadata, it has no limitation on the number or size of data objects in the collection, giving great flexibility for big data, and it provides high-performance I/O with a rich set of integrated performance features.

### Rendering

As the *FAConstructor* allows to generate fiber objects interactively, an efficient rendering of the data is crucial. Therefore, a new frame is only rendered when the user interacts with the fiber objects by rotating or translating them, allowing fluent interaction. Rendering is realized with the *OpenGL* 3.3 framework of *Qt5* [4, 25] which enables the use of widgets for applying options and generating fibers, while rendering is done in a single central widget. In addition, *OpenMP* [22] can be used optionally for several tasks granting performance improvements. Fiber objects and individual fibers are approximated by cylindrical shapes using the defined coordinates and radii. As circular objects cannot be rendered without approximation, they are represented by *n*-sided regular polygons. Even when displaying a large amount of fibers, the tool maintains a sufficient frame rate: The parameter *n* is dynamically adjusted to ensure a frame rate above 30. In addition, double-buffered *V-Sync* is used to reduce stutter which might occur when encountering a fluctuating frame rate. Nonvisible fiber objects are culled out using the *Cohen–Sutherland* algorithm [8]. As the fiber object trajectory is a line until drawn in the *OpenGL* shaders, this algorithm can be applied. The user is able to interact with the rendering area by moving, rotating, and scaling the shown content. When losing focus of the current object, the user can reset the view, displaying the whole object in the center. It is also possible to display different orthogonal views of the model and walk through the model using keyboard shortcuts. The user is able to hide certain fiber objects or whole groups by deselecting the checkboxes next to their corresponding labels (cf. Fig. 2b). This helps to keep an overview and improves performance.

Fiber bundles are colored according to their respective orientation in three-dimensional space, using either an *RGB* (red–green–blue) or an *HSV* (hue–saturation–value) color map which are also used to visualize 3D-PLI data shown in Fig. 1. In the right bottom corner of the rendering area, a color sphere displays the orientation of the fiber objects. The sphere changes when the user rotates the model. To distinguish individual fibers, it is also possible to assign each fiber bundle a randomized color based on the fiber index.

### Performance measurements

To ensure that the user can work fluently with a large number of fiber points in *FAConstructor*, performance optimizations were implemented as mentioned in “Rendering” section and tested on the generation and rendering of fiber objects. Performance measurements were performed using a PC with an Intel Core i5-3570 processor paired with an NVIDIA GTX 1070 and 16 GB RAM running Ubuntu 18.04.

Figure 3 shows the measured performance of the program. The corresponding datasets were created with the following parameters:

1 $$\begin{aligned}&(A), (C) : x(t) = t, \quad y(t) = t,\nonumber \\&z(t) = t, \quad r(t) = t, \quad t \in [0, 1, \ldots , N] \end{aligned}$$

2 $$\begin{aligned}&(B) : x(t) = \sin (t), \quad y(t) = \cos (t), \nonumber \\&z(t) = t, \quad r(t) = 1, \quad t \in [0, 1, \ldots , N] \end{aligned}$$

where *x*(*t*), *y*(*t*), *z*(*t*) are space coordinates and *r*(*t*) is the radius of the fiber object at this position. The functions describe fiber objects consisting of $N-1$ segments. This allows to conduct performance measurements as a function of the number of objects.

The test procedures to generate Fig. 3a, b, d consisted of starting the program, creating or loading the corresponding data points, and measuring the time or frame rate. Afterward, the program was closed to avoid possible influence on the following iteration. This procedure was repeated ten times for each data point. Measuring the performance when filling the fiber bundles was done by creating a dataset containing *n* fiber objects with 1000 data points each using Eq. 1. The fiber objects were filled with fibers with a distance of $d = 0.2$ and a radius of $r = 0.1$, yielding 21 fibers. The measured time of 20 separate runs was taken and averaged. As the program can use *OpenMP* for parallelization, both variants were measured individually.

As seen in Fig. 3a–c, the program tends to scale linearly with the produced amount of data points. The usage of *OpenMP* shows a speedup of 1.21 to 2.1 depending on the measured part of the program. The frame rate in (D) maintains the 30 fps target when the amount of fiber points lies below 1.6 million. This is due to the insufficient performance of the graphics card to display all data points simultaneously. Even when rendering around 60 million data points, 11 GiB RAM and 2.3 GiB video memory are consumed, indicating that neither is a limiting factor. When rendering, the CPU usage stays around 0% to 10%.

## Usecase: Fiber Cup phantom

The *Fiber Cup* phantom introduced by Fillard et al. [7] was used for qualitative and quantitative evaluation of tractography methods in dMRI. It was inspired by nerve fiber configurations in a coronal human brain section, including regions with fiber crossings, fanning, and bending. Both the reconstruction of crossing and that of fanning fibers represent a challenge for 3D-PLI. The reconstructed phantom used for our simulations was similar to the original *Fiber Cup* phantom, only deviating slightly in the crossing angles between the fiber bundles.

**Fig. 4:**
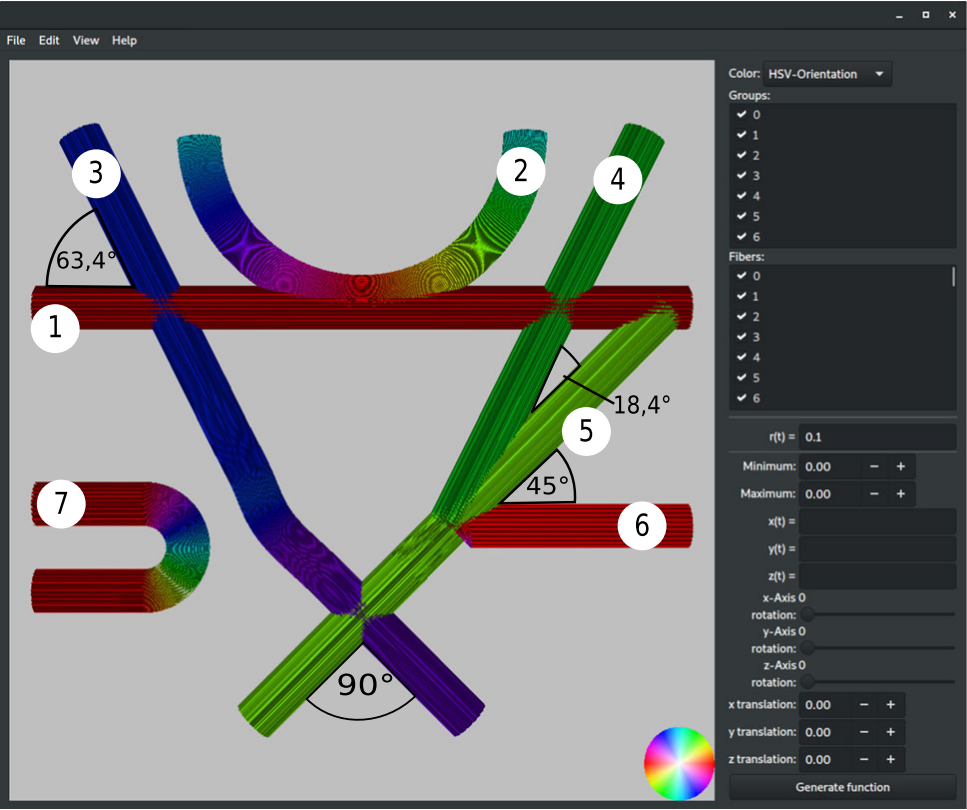
*Fiber Cup* phantom filled with fibers. The different fiber bundles (numbered from 1 to 7) were generated as described in “Usecase: Fiber Cup phantom” section

The *Fiber Cup* phantom was built using parametric functions as well as user-defined fiber points with cubic spline interpolation. When generating the *Fiber Cup* phantom, some of the fiber objects partially overlap. This leads to a higher density of fibers when filling the objects. Fiber bundles 4 to 6, for example, merge into one bundle (see Fig. 4), resulting in a three times higher fiber density as compared to the transverse bundle 3.

The fiber objects were filled with equidistant seed points on a triangular grid (see “Generation of fiber objects” section), yielding 10,149 fibers in total. The fibers have diameters of $1.5\,\upmu \hbox {m}$ and lie $1.5\,\upmu \hbox {m}$ apart, and the diameter of the fiber bundles is $120\,\upmu \hbox {m}$. The result is shown in Fig. 4.

**Fig. 5:**
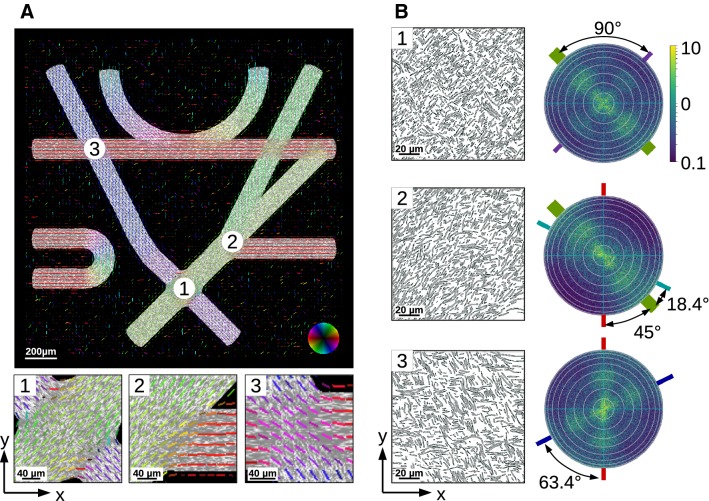
**a** Vector field obtained from *simPLI* simulations of the collision-free *Fiber Cup* phantom, overlaid with the employed fiber model. The orientation of the vectors are color-coded. Visualization has been realized with in-house developed software [24]. **b** FDTD simulations of the three selected regions (fiber crossings) in the collision-free *Fiber Cup* phantom shown in (**a**). The images on the left show a cross section (*xy*-plane) through the middle of the simulated fiber volume ($128 \times 128 \times 60\,\upmu \hbox {m}^3$). The images on the right show the corresponding scattering patterns (intensity per wave vector). The white circles indicate steps of $10^{\circ }$, from $0^{\circ }$ (center) to $90^{\circ }$ (outer circle). The straight colored lines around the scattering patterns indicate the axes perpendicular to the fiber bundles in the phantom (colors were chosen according to the colors of the fiber bundles shown in **a**)

However, as two distinct nerve fibers in real brain tissue do not occupy the same point in space in a crossing situation, the generated fiber configurations were checked for such unrealistic collisions and solved using an additional in-house developed software [15] before performing the imaging/measurement simulations. Depending on the initial objects’ fraction of overlap, the density of the fibers can change during this process. Most likely, this results in a decrease in density, since the fiber objects are designed as rigid objects. In the *Fiber Cup* example, the fiber densities within the intersecting regions 1 to 3 in Fig. 5 decreased by $29\%(1), 25\%(2)$, and $3\%(3)$, respectively.

### Three-dimensional polarized light imaging (3D-PLI)

3D-PLI is a postmortem imaging technique for unstained histological brain sections. Due to the birefringence (optical anisotropy) of the nerve fibers, the state of polarization changes when light passes through brain tissue. In 3D-PLI, this change of polarization is measured with a polarimetric setup (polarizer, specimen, quarter-wave retarder, analyzer), allowing to determine the spatial orientations of the nerve fibers with micrometer resolution. The measurement and signal analysis of 3D-PLI have been described in detail by Axer et al. [2]. As the investigated brain sections are about $60\,\upmu \hbox {m}$ thick, each measured volume element contains several nerve fibers. 3D-PLI extracts only the predominant fiber orientation, and the orientations of individual nerve fibers within deep white matter structures can therefore not be determined. Inhomogeneous nerve fiber structures, like crossing fibers or regions with varying fiber densities, influence the measured birefringence signal and may lead to misinterpretations in the reconstructed fiber orientations.

### Simulation methods

*Matrix calculus (simPLI)* The in-house developed software *simPLI* [6] computes the change of polarization of a light beam when passing through a brain tissue sample. The light beam is modeled by Stokes vectors, the optical elements of the polarimeter and the brain tissue by Müller matrices [21]. The birefringence of the nerve fibers is modeled by optical retarder matrices, with the retarder axis oriented along the fiber axis [16]. To account for the imaging system, the image is downsampled and blurred with a Gaussian function with $\sigma = 0.714\,\Delta {x}$ as described by Menzel et al. [16]. Finally, noise is modeled according to Schmitz et al. [23]. The resulting signals are analyzed using the *ROFL* algorithm [23], resulting in fiber orientation maps.

*Finite-Difference Time-Domain (FDTD) simulation * To study more complex light–matter interactions like scattering and interference, *Finite-Difference Time-Domain* (FDTD) simulations are used. With FDTD simulations, it is possible to study light transmitted through artificial nerve fiber models as well as scattering of light [18, 19]. The propagation of the electromagnetic light wave through the sample was computed by $\textit{TDME3D}^{{\mathrm{TM}}}$, a massively parallel three-dimensional Maxwell Solver based on a conditionally stable FDTD algorithm [20, 28]: The electromagnetic field components are computed numerically by discretizing space and time and approximating the spatial and temporal derivatives in Maxwell’s curl equations by second-order central differences. More details about the algorithm can be found in Menzel et al. [17].

### Simulation results

*Matrix calculus (simPLI)* The collision-free *Fiber Cup* phantom was used as input for *simPLI* (see “Simulation methods” section). The fibers were modeled as homogeneous materials with an absorption of $\mu = 5\hbox { mm}^{-1}$, and the birefringence was modeled with a strength of $\Delta {n} = 0.001$ and one axis of anisotropy oriented along the longitudinal fiber axis [16]. For the simulation, a section of $60\,\upmu \hbox {m}$ thickness from the mid-plane of the *Fiber Cup* phantom was used, discretized into voxels of $1\,\upmu \hbox {m}$ side length. The simulations were performed with wavelength $\lambda = 525\hbox { nm}$, ingoing light intensity $I_{{0}} = 26{,}000$, and image pixel size $\Delta {x} = 20\,\upmu \hbox {m}$.

Figure 5a shows the resulting fiber orientations as a vector field, overlaid with the original fiber model. In regions with straight fiber bundles, the fiber orientations are in good agreement with the underlying fiber structure. In crossing regions, however, some fiber orientations are misinterpreted. In region (3), where both crossing fiber bundles contain the same amount of fibers, the resulting vector field shows the average orientation of the two underlying fiber bundles (pink). If fiber tractography, algorithms were applied to the vector field, and the crossing fibers ($$\times $$) could be misinterpreted as kissing fibers ($$>
<$$). In region (1), the resulting vector field is dominated by the orientation of the fiber bundle (green) that consists of three bundles (nos. 4, 5, and 6, see Fig. 4). The orientation of the other fiber bundles (purple) is not visible in the resulting vector field so that the crossing of the fiber bundles is not detected. In region (2), the three fiber bundles (4, 5, and 6) join each other. Bundles 4 and 5 (green) have similar orientations and are already indistinguishable when they merge with bundle 6 (red). The resulting orientation vectors of bundle 6 assimilate with the orientation vectors of the other two bundles. The original course of bundle 6 in the crossing regions is not visible in the resulting vector field.

*Finite-Difference Time-Domain (FDTD) simulation * The three selected regions of the *Fiber Cup* phantom in Fig. 5a were also studied with FDTD simulations to investigate the scattering of light. For this purpose, a volume of $128 \times 128 \times 60\,\upmu \hbox {m}^3$ was selected in each region and the propagation of the light wave through the sample was simulated as described in the “Simulation methods” section. The simulations were performed for uniaxial perfectly matched layer absorbing boundaries with a thickness of $1\,\upmu \hbox {m}$, a Yee mesh size of 25 nm, a duration of 400 periods, a Courant factor of 0.8, and normally incident light with 550 nm wavelength (modeling the polarizing microscope) [18, 19]. All tissue components of the sample (fibers and surrounding tissue) were modeled by dielectric materials with real refractive indices (neglecting absorption). Each fiber was modeled by an inner axon and a surrounding myelin sheath with two layers, as described by Menzel et al. [18]. The simulations were performed on the supercomputer *JURECA* [11] at Forschungszentrum Jülich GmbH, Germany. Figure 5b shows the resulting scattering patterns, i. e. the intensity per wave vector, for the three selected regions. The scattering patterns reveal the underlying fiber structure and crossing angles of the fiber bundles. The light is always scattered under angles perpendicular to the principal axis of the corresponding fiber bundle. For reference, the perpendicular axes are indicated by straight colored lines around the scattering patterns. (Colors were chosen according to the colors of the fiber bundles shown in Fig. 5a.) In contrast to *simPLI*, the FDTD simulations allow to resolve individual fiber orientations also in regions with crossing fibers. The determined angles correspond to the angles of the *Fiber Cup* phantom (cf. Figs.  4 and 5b). Bundles with a larger amount of fibers (regions (1), (2), in green) cause stronger scattering than bundles with a smaller amount of fibers.

## Discussion and conclusion

We have introduced the *FAConstructor* tool enabling the interactive generation of synthetic nerve fiber models both in an accurate and efficient way. The fast generation of many different fiber models is a prerequisite for imaging simulation tools to study physical effects in virtual brain tissue. Functionalities like modification of fiber objects (translation, rotation, scaling, filling), grouping of several fiber objects (e. g. fibers to a fiber bundle) and successive generation of building blocks (as groups) up to the final model allow a fast generation and easy (re)production of phantoms. By defining the radius as a function along the fibers, it is also possible to build multiple layers (e. g. myelin with nodes of Ranvier) around an axon.

Evaluating the performance of the developed tool shows that most processes scale almost linearly with the amount of data points used to describe the fiber models. When working with simple, unfilled objects, the program guarantees a smooth interaction proven by the performance measurements shown. Models with less than 1.6 million data points retain a frame rate of more than 30 frames per second. Assuming a constant diameter of nerve fibers in the human brain of $0.5\,\upmu \hbox {m}$ [12] and, for a high detailed model, a step size of about $1\,\upmu \hbox {m}$, the here used hardware could render a stable frame rate for a filled cubic volume of about $80\,\upmu \hbox {m}^{3}$. It is therefore also capable of visualizing a high detailed model for the FDTD simulation. For building larger models, the usage of hiding groups can improve the rendering temporarily, where the final model can still be rendered when necessary.

As the 3D-PLI simulations only model sections of brain tissue with $60\,\upmu \hbox {m}$ thickness, more than 1.6 million data points are rarely necessary, which guarantees a stable and fluent workflow. For comparison, the newly generated *Fiber Cup* phantom contains 426,300 data points after filling and the original fiber bundle contains only 290 data points. This indicates that even large fiber models can be rendered on similar hardware. However, to create even more detailed and/or larger brain tissue models beyond pure white matter phantoms (e. g. cerebral cortex phantoms including astrocytes, glial cells, and neuropil), a supercomputing environment is required. First tests of the *FAConstructor* on one visualization node of the supercomputer *JURECA* [11] at the Supercomputing Center at Forschungszentrum Jülich GmbH, Germany, showed promising performance. This aspect will be addressed in future studies.

The applicability of *FAConstructor* was demonstrated for two different simulation approaches using the example of the *Fiber Cup* phantom. The generation of the phantom took around ten minutes for an instructed user, indicating the user-friendly environment of *FAConstructor*. Since the tool can be used to generate large-scale fiber models with small-scale detail, it is suited for both large-scale *simPLI* and small-scale FDTD simulations. It will help to generate the models quickly and, if necessary, to modify them accordingly so that the underlying fiber structure can be tested from experimental data in an appropriate time.

In order to enable faster and more intuitive changes to the models, mouse interactions with the scene need to be implemented. This would allow more intuitive modification of fiber objects, such as moving fiber points or changing entire objects or groups. Another improvement would be the visualization of the 3D-PLI data or more general gray-scale and RGB images, or three-dimensional vector data. By this means, it would be possible to visualize the generated structures with the experimental results and the resulting simulation data. Therefore, not only the construction process of the models would be improved, but also the interpretation and analysis process of the simulation results in relation to the models used. A future implementation could also provide an interface for plug-ins that would allow algorithms, analyses, or even the simulation process to be run. Since a model without colliding fibers is very important for 3D-PLI simulations, the colliding solving process could be executed and visualized step-by-step.

Due to the general definition of fibers as geometric objects defined by points and radii, other neuroimaging simulation techniques like dMRI are also capable of using the models generated by *FAConstructor*. Since the technological and analytical methods in dMRI improve continuously, highly detailed microstructured models are required for simulations to address the impact of underlying neuronal components on the diffusion behavior [9].

In conclusion, we have implemented a user-friendly and efficient tool that enables a fast generation of complex and realistic synthetic nerve fiber configurations (i. e. numerical brain tissue phantoms) ideally suited for the simulation of neuroimaging techniques like 3D polarized light imaging. The generated fiber bundles can be visualized simultaneously and edited interactively. This ultimately enables the production of application-specific well-defined ground truth datasets.

## Electronic supplementary material

Below is the link to the electronic supplementary material.

Supplementary material 1 (mp4 92543 KB)
